# Synthesis of a New Titanate Coupling Agent for the Modification of Calcium Sulfate Whisker in Poly(Vinyl Chloride) Composite

**DOI:** 10.3390/ma9080625

**Published:** 2016-07-28

**Authors:** Wenjin Yuan, Yunhua Lu, Shiai Xu

**Affiliations:** 1 School of Chemical Engineering, Qinghai University, Xining 810016, China; ywj407024846@126.com; 2 School of Materials Science and Engineering, East China University of Science and Technology, Shanghai 200237, China; 18521399954@163.com

**Keywords:** poly(vinyl chloride) composites, calcium sulfate whisker, coupling agent, surface treatment, interface

## Abstract

A new titanate coupling agent synthesized from polyethylene glycol (PEG), isooctyl alcohol, and phosphorus pentoxide (P_2_O_5_) was used for the modification of calcium sulfate whiskers (CSWs) and the preparation of high-performance CSW/poly(vinyl chloride) (PVC) composites. The titanate coupling agent (sTi) and the modified CSWs (sTi–CSW) were characterized by Fourier transform infrared (FTIR) spectroscopy, and the mechanical, dynamic mechanical, and heat resistant properties and thermostability of sTi–CSW/PVC and CSW/PVC composites were compared. The results show that sTi–CSW/PVC composite with 10 wt. % whisker content has the best performance, and its tensile strength, Young’s modulus, elongation at break, break strength, and impact strength are 67.2 MPa, 1926 MPa, 233%, 51.1 MPa, and 12.75 KJ·m^−2^, with an increase of 20.9%, 11.5%, 145.3%, 24.6%, and 65.4% compared to that of CSW/PVC composite at the same whisker content. As the whisker content increases, the storage modulus increases, the Vicat softening temperature decreases slightly, and the glass transition temperature increases at first and then decreases.

## 1. Introduction

Poly(vinyl chloride) (PVC) is one of the most widely used general-purpose plastics, with a number of advantageous properties, such as good flame retardancy, corrosion resistance, and wear resistance [1]. It is generally classified into two types: rigid PVC with high strength and stiffness, and soft PVC with low-molecular-weight plasticizers that can greatly weaken the interactions between PVC chains [2]. The rigid PVC also has some intrinsic disadvantages, such as low impact strength and low resistance to microcrack propagation, thus severely limiting its applications as a high-performance structural material [3]. A variety of fillers have been incorporated into the PVC matrix to improve its performance (e.g., toughness, modulus, permeability, and heat resistance) and to reduce the production cost [4]. These fillers can be roughly divided into four types, according to their shapes: one-dimensional fillers (e.g., natural fibers [5,6], carbon nanotubes [7,8,9], and whiskers [10,11,12]), two-dimensional fillers (e.g., montmorillonite [13,14], graphene (G) [15,16,17,18], and graphene oxide [18]), three-dimensional fillers (e.g., CaCO_3_ particles [19,20], SiO_2_ particles [21,22], and TiO_2_ particles [23,24]), and the compounds of different dimensional fillers (e.g., Ag/G [17] and TiO_2_/G [25]). Calcium sulfate whisker (CSW) is a fiber-shaped single crystal with many desirable properties, such as high strength, high stiffness, and low cost [26], making it a desirable filler for the reinforcement of polymer composites.

However, the incorporation of most fillers, such as multiple-walled carbon nanotubes [27], CaCO_3_ particles [28], and nano-TiO_2_ particles [24], into the PVC matrix results in a decrease of the tensile strength of the composite. This is mainly because the interfacial interactions between the fillers and the polymer matrix are poor, and thus the stress applied to the composite cannot be transmitted sufficiently from the matrix to the fillers, leading to premature rupture of the composite [29]. Thus, the surface of the fillers needs to be modified to improve their wettability and adhesion with the polymer matrix [30]. This can be achieved with the use of appropriate coupling agents such as silane coupling agents [31,32], titanate coupling agents [33], and fatty acids [34]. However, a potential drawback of these commercial coupling agents is that they only have either polar or nonpolar groups on their chains.

The main purpose of this study is to synthesize a new titanate coupling agent having both polar and nonpolar groups, used for the modification of CSW. The polar groups have a strong interaction with the PVC matrix and thus can improve the interfacial interaction between CSW and the matrix, while the nonpolar groups can act as flexible chains to improve the toughness of the composite. Titanate coupling agent modified CSW (sTi–CSW)/PVC composite shows better tensile strength, stiffness, elongation at break, break strength, and impact strength than CSW/PVC composite. The toughness of PVC is significantly improved without a significant decrease of tensile strength.

## 2. Results and Discussion

### 2.1. Fourier Transform Infrared (FTIR) Spectra of the Titanate Coupling Agent and sTi–CSW

The structural formula of the target product is as follows:

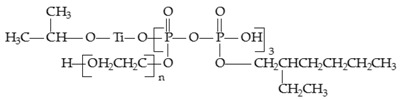
(1)

**Figure 1:**
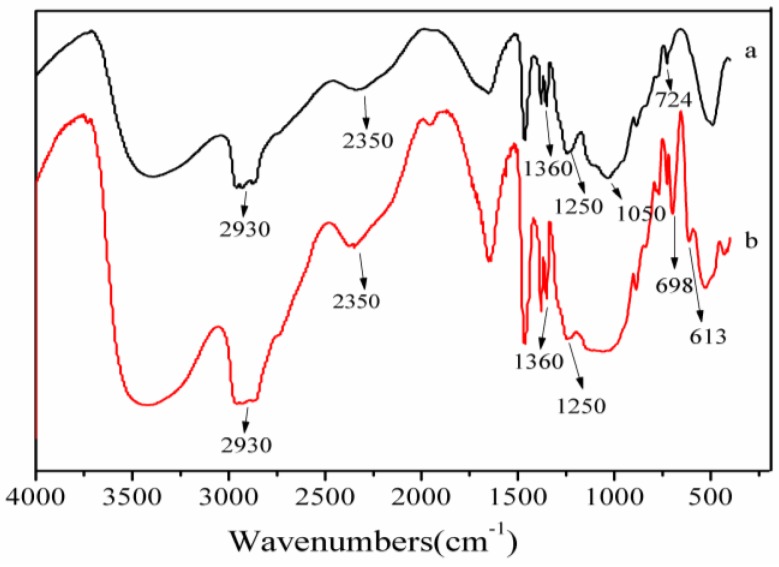
Fourier Transform Infrared (FTIR) spectra of (**a**) pyrophosphate and (**b**) self-synthesized titanate coupling agent.

Figure 1 shows the FTIR spectra of the intermediate product (pyrophosphate) and the target product (titanate coupling agent). The absorption band at 724 cm^−1^ is assigned to the absorption of the CH_2_CH_2_O groups of polyethylene glycol (PEG), and the absorption intensity increases as the chain length increases. The absorption band at 2930 cm^−1^ is assigned to the stretching vibration of the CH_3_ and CH_2_ groups in isooctyl alcohol and that of the CH_2_ groups in PEG, which is most indicative of the presence of PEG. The absorption band at 1360 cm^−1^ is assigned to the rocking vibration of CH_2_. The wide absorption band at 2564–2000 cm^−1^ is assigned to the characteristic absorption of P–O–H (broad peak at 2350 cm^−1^). The absorption band of pyrophosphate is between 1310 and 1210 cm^−1^, so the peak at 1250 cm^−1^ may be the absorption peak of that group. The peak at 1050 cm^−1^ is assigned to the characteristic absorption of C–O. The new absorption peaks at 613 and 698 cm^−1^ in line b are the absorption bands of Ti–O. These results imply that a new titanate coupling agent with both PEG and isooctyl alcohol segments has been successfully synthesized.

**Figure 2:**
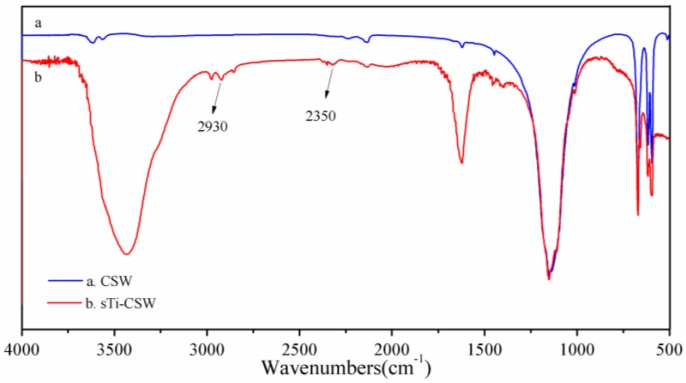
FTIR spectra of (**a**) calcium sulfate whisker (CSW) and (**b**) titanate coupling agent modified CSW (sTi–CSW).

Figure 2 shows the FTIR spectra of CSW and sTi–CSW. When compared with CSW, two new absorption peaks are observed at 2930 and 2350 cm^−1^ in sTi–CSW, which are the characteristic absorption bands of the titanate coupling agent, and thus indicate the successful modification of CSW.

**Figure 3:**
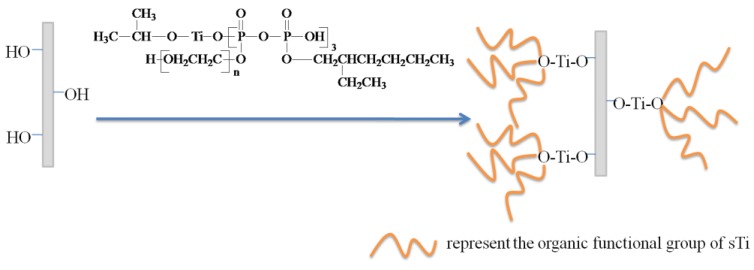
The modification mechanism of sTi–CSW.

Figure 3 shows the modification mechanism of sTi–CSW. The alkoxy groups of the titanate coupling agent can easily hydrolyze and react with the hydroxyl groups of CSWs. Thus, the polar groups are grafted to CSWs, which can interact with polar PVC and thus improve the properties of the composite.

### 2.2. Mechanical Performance

**Figure 4:**
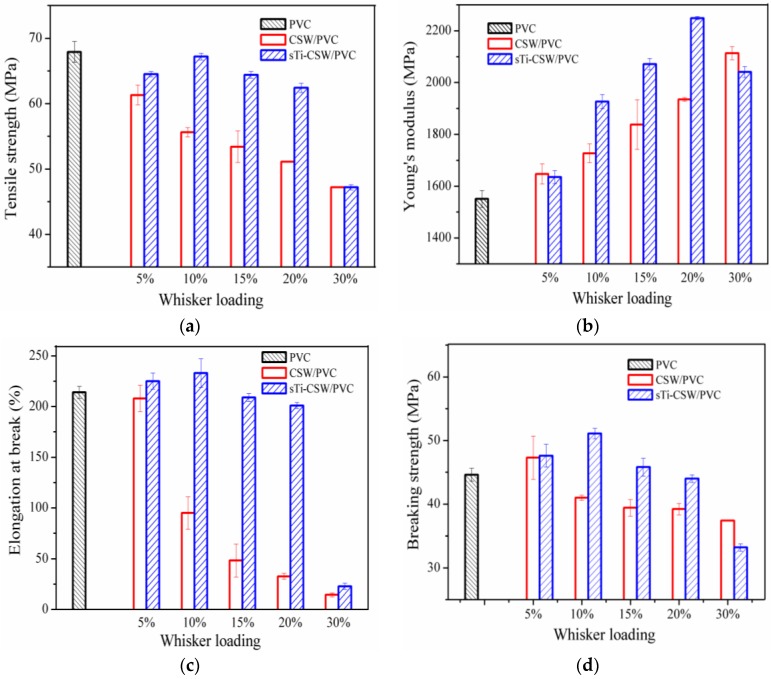
Mechanical properties (**a**) Tensile strength; (**b**) Young’s modulus; (**c**) Elongation at break; (**d**) Breaking strength of CSW/PVC and sTi–CSW/PVC composites with various filler contents.

Figure 4 shows that as the whisker content increases, the tensile strength of the CSW/PVC composite decreases, whereas that of sTi–CSW/PVC composite increases at first and then decreases. The tensile properties of sTi–CSW/PVC composite—especially the elongation at break—are obviously better than that of CSW/PVC composite. The elongation at break of CSW/PVC composite decreases rapidly with the increase of the whisker content, while that of sTi–CSW/PVC composite remains at a relatively high level with the increase of the whisker content up to 20 wt. %, but decreases significantly with further increase of the whisker content to 30 wt. %. The sTi–CSW/PVC composite has the best performance at 10 wt. % whisker content. In this case, its tensile strength, Young’s modulus, elongation at break, and break strength are 67.2 MPa, 1926 MPa, 233%, and 51.1 MPa, with an increase of 20.9%, 11.5%, 145.3%, and 24.6% compared with that of CSW/PVC composite at the same whisker content. However, the tensile properties of sTi–CSW/PVC composite decrease significantly at 30 wt. % whisker content. This may be because there is a percolation threshold of the filler content for the composite [35], exceeding which the fillers are more likely to collide with each other, thus leading to fracture or crumble of these fillers, and consequently the decrease of the reinforcing and toughening effects [2]. As a result, the mechanical properties of the composite decrease dramatically. In general, sTi–CSW can obviously improve the tensile properties of the composite. This may be due to the fact that the polar groups in the organic chain of the coupling agent have strong interactions with the PVC matrix, leading to the improvement of the interfacial bonding between the matrix and the whiskers [36,37], and that the flexible segments (the alkane and ether groups) of the coupling agent can act as a toughening agent, leading to the improvement of the elongation at break. 

**Figure 5:**
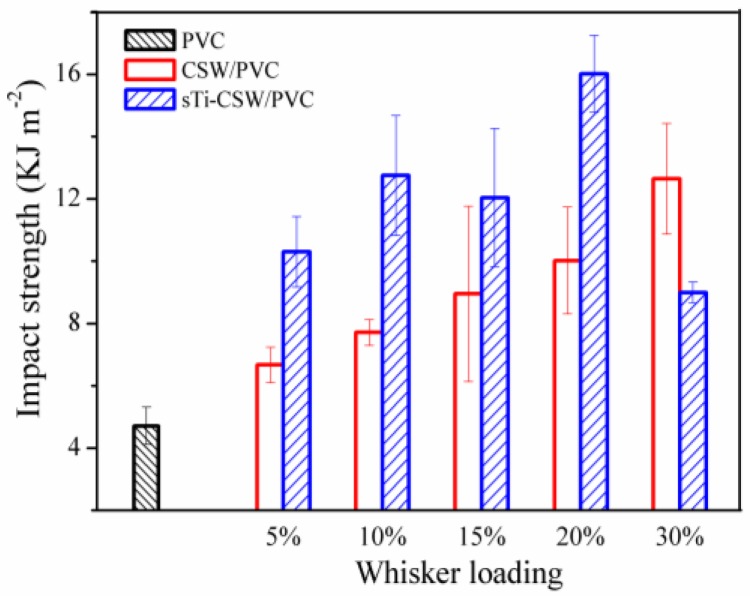
Impact strength of CSW/PVC and sTi–CSW/PVC composites with various filler contents.

Figure 5 shows the Izod impact strength of CSW/PVC and sTi–CSW/PVC composites with different CSW contents. It can be seen that the impact strength is increased from 7.71 KJ·m^−2^ for CSW/PVC to 12.75 KJ·m^−2^ for sTi–CSW/PVC with 10 wt. % CSWs. This may also be due to the combined effects of the flexible segments and the strong interfacial interaction. The impact strength of sTi–CSW/PVC composite also decreases rapidly at 30 wt. % whisker content, which is consistent with the tensile properties.

### 2.3. Interfacial Morphology

**Figure 6:**
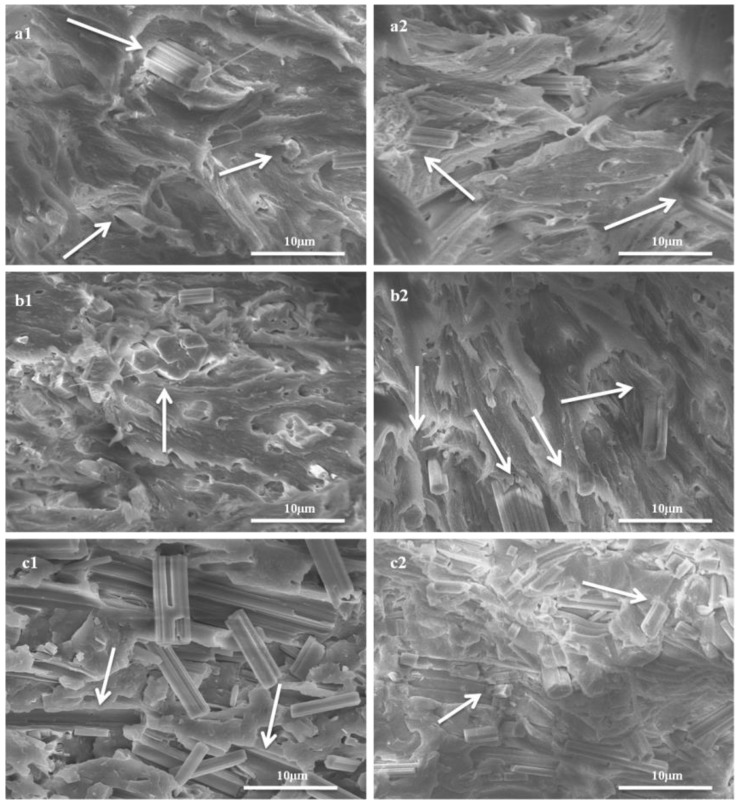
SEM images of the tensile fracture surfaces of CSW/PVC with (**a1**) 5 wt. %; (**b1**) 10 wt. % and (**c1**) 30 wt. % whisker (Thanks for the permission from J. Mater. Sci. Technol.); and sTi–CSW/PVC with (**a2**) 5 wt. %; (**b2**) 10 wt. % and (**c2**) 30 wt. % whisker.

Figure 6 shows the SEM images of the tensile fracture surfaces of CSW/PVC [38] and sTi–CSW/PVC composites with 5, 10, and 30 wt. % CSWs. For CSW/PVC composite, pullouts and gaps between whiskers and PVC matrix are clearly observed, and the whiskers are more likely to agglomerate as their content increases. However, for sTi–CSW/PVC composite, the PVC matrix adheres tightly to the whiskers at 5 and 10 wt. % whisker content. This may also be due to the strong interaction between the polar groups of the coupling agent and the PVC matrix, which can improve the interfacial properties of the composite [12]. Although the interfacial interaction between whiskers and matrix is still good at 30 wt. % whisker content, the whisker size decreases due to collisions during the mixing process that may lead to the fracture of whiskers.

### 2.4. Dynamic Mechanical Properties

**Figure 7:**
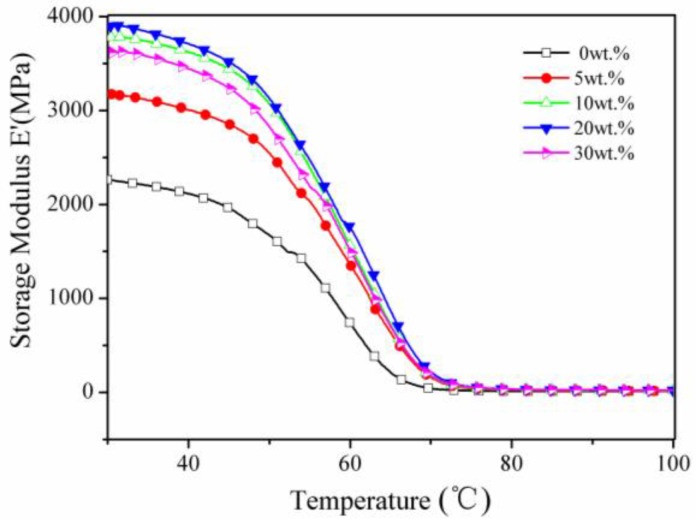
Storage modulus as a function of temperature for sTi–CSW/PVC composites with different whisker contents.

**Figure 8:**
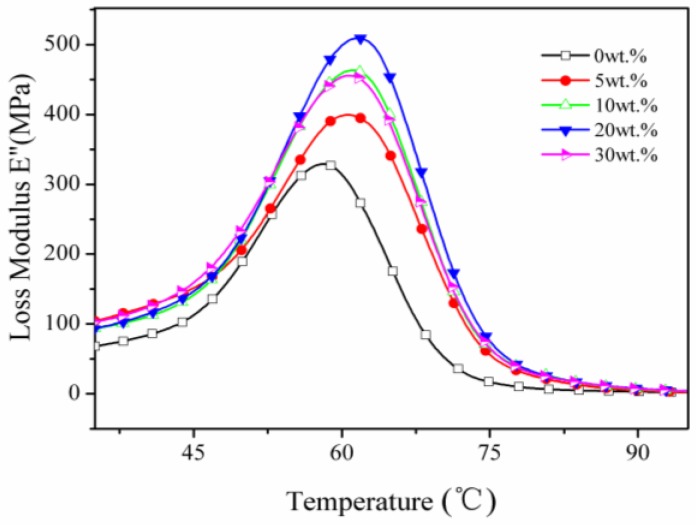
Loss modulus as a function of temperature for sTi–CSW/PVC composites with different whisker contents.

**Figure 9:**
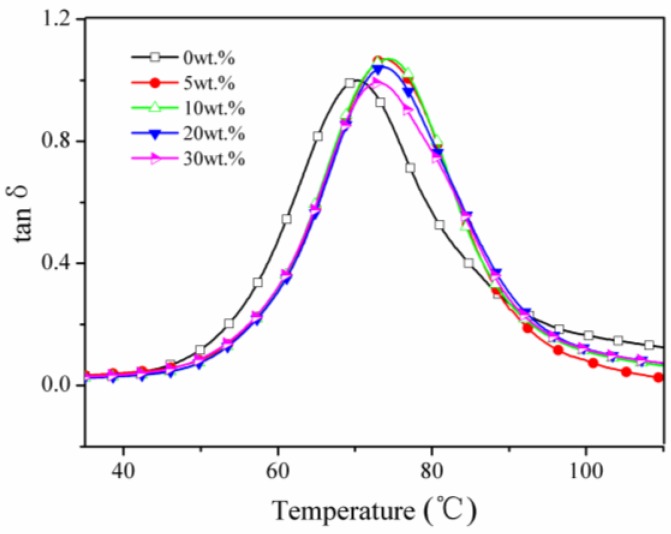
Tanδ as a function of temperature for sTi–CSW/PVC composites with different whisker contents.

The thermomechanical properties of sTi–CSW/PVC composite with different whisker contents—including storage modulus (E’), loss modulus (E”), and tangent δ (tan δ)—were determined by dynamic mechanical analysis (DMA), and the results are shown in Figure 7, Figure 8 and Figure 9, respectively. Figure 7 shows that at low temperatures, the storage modulus of sTi–CSW/PVC composite increases with increasing whisker content, except at 30 wt. % whisker content. Besides, it is much higher than that of CSW/PVC composite at the same whisker content (Table 1), indicating that the titanate coupling agent can efficiently improve the interfacial interaction between whiskers and PVC matrix [39]. This allows the whiskers to effectively restrict the deformation of the matrix, thus resulting in the improvement of the performance of the composite [40,41].

Figure 8 and Figure 9 show that the peak temperature of the composite increases at first and then decreases with the increase of the whisker content. The glass transition temperature of sTi–CSW/PVC and CSW/PVC composites are listed in Table 2. For sTi–CSW/PVC composite, the glass transition temperature reaches a maximum at 10 wt. % whisker content, and then decreases with further packing of whiskers, probably due to the presence of both polar organic groups and nonpolar flexible alkane chains in the coupling agent. The polar groups have strong interactions with the PVC matrix, which can prevent the PVC matrix from deforming at high temperatures and improve the glass transition temperature of the composite. However, the alkane chains and the ether groups are flexible, which can decrease the glass transition temperature.

### 2.5. Heat Resistance Property

**Figure 10:**
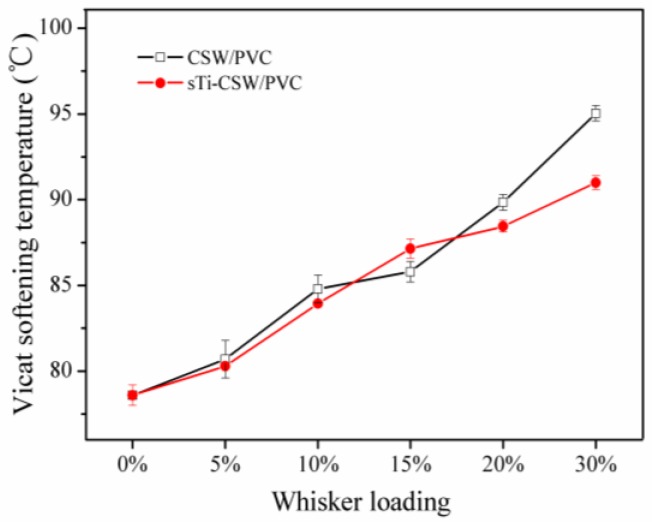
Vicat softening temperature (VST) for CSW/PVC and sTi–CSW/PVC composites with various filler contents.

Vicat softening temperatures (VST) reflect the moving ability of chain segments. The more difficult it is for the chain segments to move, the higher the VST will be [42]. Figure 10 shows the VST of CSW/PVC and sTi–CSW/PVC composites with various filler contents. It can be seen that as the whisker content increases, the VST of both CSW/PVC and sTi–CSW/PVC composites increases, because these whiskers can effectively restrict the mobility of the PVC segments due to their large length-to-diameter ratio [43]. However, the VST of the sTi–CSW/PVC composite is slightly lower than that of the CSW/PVC composite, due to the flexible chains in the coupling agent.

### 2.6. Thermal Properties

**Figure 11:**
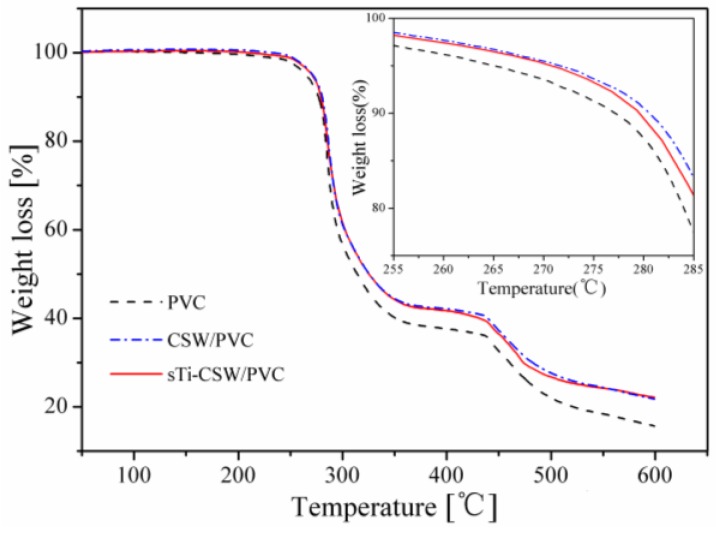
Thermogravimetric analysis (TGA) curves of PVC and its composites.

**Figure 12:**
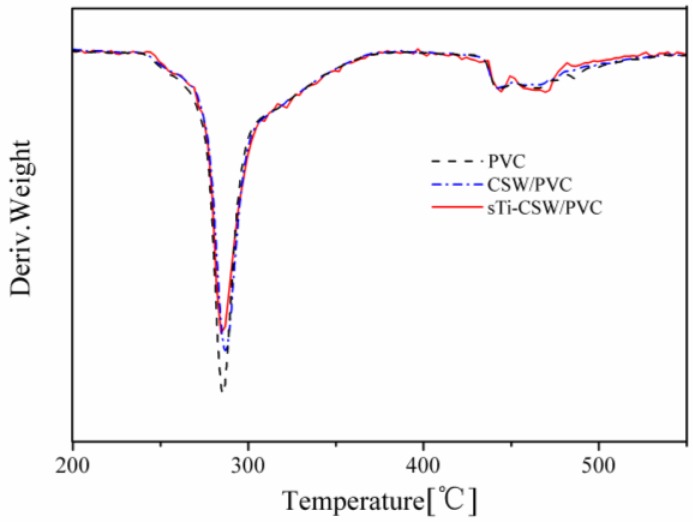
Differential thermogravimetry (DTG) curves of PVC and its composites.

The thermogravimetric analysis (TGA) and differential thermogravimetry (DTG) curves of pristine PVC, CSW/PVC, and sTi–CSW/PVC composites are shown in Figure 11 and Figure 12, respectively. It shows that there are two weight loss stages for pristine PVC, CSW/PVC, and sTi–CSW/PVC composites. The first decomposition stage starts at about 276 °C and ends at about 298 °C, which can be attributed to the dehydrochlorination that may induce double bonds along the polymer chain and lead to conjugated polymer chains [44]. However, the sample becomes thermally stable again at 298–435 °C due to the formation of conjugated double bonds after HCl evolution [45]. A second decomposition stage occurs at 435–489 °C, which corresponds to the polyacetylene cracking (scission of covalent and multiple bonds). A stable residue (i.e., carbon black) is formed at temperatures greater than 489 °C [46].

The temperatures of onset decomposition (T_onset_), rapidest decomposition (T_rpd_), and 50% weight loss residue (T_50_) are summarized in Table 3. It can be seen that the T_onset_ and T_rpd_ of sTi–CSW/PVC composite are quite close to that of CSW/PVC composite and pristine PVC, indicating no significant improvement of thermal degradation in CSW/PVC and sTi–CSW/PVC composites [12]. The TGA curve of sTi–CSW/PVC composite is almost coincident with that of CSW/PVC composite, and their T_50_ values are higher than that of pristine PVC, indicating that the thermal stability of both sTi–CSW/PVC and CSW/PVC composites are improved to a certain degree [46,47]. However, the thermal stability of the composite cannot be further improved by sTi–CSW.

## 3. Materials and Methods

### 3.1. Materials

P_2_O_5_ (analytically pure), isooctyl alcohol (chemically pure) and dichloroethane (analytically pure) were purchased from Shanghai Lingfeng Reagent Co., Ltd. (Shanghai, China). Titanium isopropoxide was purchased from Aladdin Reagent Co., Ltd. (Shanghai, China). PEG 200 (chemically pure) was purchased from Sinopharm Chemical Reagent Co., Ltd. (Shanghai, China). PVC (SG-5) was purchased from Dongguan Dansheng Plastic Materials Co., Ltd. (Dongguan, China). CSWs were purchased from Shanghai Fengzhu Trading Co., Ltd. (Shanghai, China). Organic tin, dioctyl phthalate (DOP), glyceryl monosterate (GMS), acrylic processing aid (ACR) and paraffin wax were commercially available, all of which were of technical grade.

### 3.2. Preparation

#### 3.2.1. Synthesis of Polar Polyether Titanate Coupling Agent

Pyrophosphate was prepared as follows: 2.1 g of P_2_O_5_ and 40 mL of dichloromethane solvent were added sequentially into a 250 mL three-necked round-bottom flask fitted with a mechanical overhead stirrer, and stirred rapidly. Then, 5.28 g of isooctyl alcohol and 4 g of PEG 200 were added slowly, the mixture was stirred at 60 °C for 2 h, and excess solvent was removed by vacuum distillation. The obtained transparent liquid was pyrophosphate. 

Then, 1.6 g of titanium isopropoxide and 30 mL of dichloromethane solvent were added sequentially into a 250 mL three-necked round-bottom flask, and 8 g of pyrophosphate was added dropwise. The temperature of the water bath was maintained at 20–55 °C. The mixture was stirred at 75 °C for 2.5 h and then heated to 90 °C to complete the reaction and to remove excess solvent. The obtained light yellow and viscid liquid was the titanate coupling agent.

#### 3.2.2. Preparation of sTi–CSW and sTi–CSW/PVC Composites

sTi–CSWs were prepared as follows: 1.5 g of sTi was pre-hydrolysed in 95 wt. % ethanol solution, and 50 g of dried CSWs were added sequentially into a 500 mL three-necked round-bottom flask fitted with a mechanical overhead stirrer. The mixture was stirred at 75 °C for 4 h, and then the obtained products were dried under vacuum at 100 °C for 2 h to remove excess water.

sTi–CSW/PVC composites were prepared as described in our previous study [12]: PVC resin (100 phr) was mixed with various contents of CSW or sTi–CSW using organic tin (2 phr) as the heat stabilizer and DOP (4 phr) as the plasticizer. GMS (0.6 phr), ACR (4 phr), and paraffin wax (0.4 phr) were then added. All PVC constitutes were mixed uniformly and then processed using a two-roll mill at 170 °C. The resultant compound was molded into rectangular sheets by compression molding at 170 °C and 10 MPa for 5 min using a plate vulcanizing press.

### 3.3. Characterization

#### 3.3.1. Fourier Transform Infrared (FTIR) Spectroscopy

The FTIR spectra of the titanate coupling agent and sTi–CSW were recorded using a Nicolet 6700 FTIR spectrometer (Thermo Fisher, New York, NY, USA) with a scan number of 32 and a resolution of 4 cm^−1^.

#### 3.3.2. Mechanical Properties

The tensile properties of CSW/PVC and sTi–CSW/PVC composites were determined on a MTS E44 universal testing machine (MTS Industrial Systems, Shenzhen, China) in accordance with ISO 527 [48], and the notched impact strength was determined on a CEAST 9050 tester (CEAST, Turin, Italy) according to ISO 179 [49]. The means and standard deviations were calculated from at least five independent tests for each sample.

#### 3.3.3. Scanning Electron Microscopy

The fracture surfaces of the tensile samples were characterized by scanning electron microscopy (SEM, S-4800, Hitachi, Tokyo, Japan). Prior to SEM observation, the fracture surfaces were coated with a thin gold layer.

#### 3.3.4. Dynamic Mechanical Analysis

The dynamic properties of CSW/PVC and sTi–CSW/PVC composites were determined on a TA Instruments Q800 (TA instruments, New Castle, DE, USA) in three-point bending mode. A single specimen of each composite type was tested at a vibration frequency of 1 Hz and heated from 25 to 140 °C at a heating rate of 3 °C/min.

#### 3.3.5. Heat Resistance Analysis

Vicat softening temperatures (VST) were measured using a VST tester (ZWK1302-B, MTS, Shenzhen, China) according to ISO 306:2004 [50]. The test load was 10 N and the heating rate was 120 °C/h.

#### 3.3.6. Thermogravimetric Analysis 

Thermogravimetric analysis (TGA) was carried out at a heating rate of 10 °C/min to 600 °C under a nitrogen atmosphere using a Netzsch STA 409PC thermogravimetric analyzer.

## 4. Conclusions

A new polar organic coupling agent having both polar and nonpolar (alkane and ether) groups has been successfully synthesized in this study. The polar groups can improve the interfacial interactions between the whiskers and the PVC matrix, while the non-polar groups can improve the toughness of the composites, thus resulting in a significant improvement of the mechanical properties of the sTi–CSW/PVC composites. The tensile strength, Young’s modulus, elongation at break, break strength, and impact strength of sTi–CSW/PVC composite with 10 wt. % whisker are 67.2 MPa, 1926 MPa, 233%, 51.1 MPa, and 12.75 KJ m^−2^, with an increase of 20.9%, 11.5%, 145.3%, 24.6%, and 65.4%, respectively, as compared with that of CSW/PVC composite with the same whisker content. There is a strong interfacial interaction between the modified whiskers and the PVC matrix. The DMA results show that the storage modulus is also obviously improved. The glass transition temperature increases at first and then decreases with the increase of the whisker content. However, no obvious improvement is observed in the heat resistant property and the thermal stability, which may be due to the flexible groups of the coupling agent. In general, the coupling agent synthesized in this study can effectively improve the toughness of PVC without significantly decreasing its tensile properties. Thus, this coupling agent may have important applications in the modification of inorganic fillers.

## Figures and Tables

**Table 1:** The storage modulus (MPa) of CSW/PVC and sTi–CSW/PVC composites at 30 °C.

Whisker Content (%)	0	5	10	20	30
CSW/PVC	2263	2760	2871	2929	3540
sTi–CSW/PVC	2263	3172	3780	3900	3616

**Table 2:** Glass transition temperature (°C) of CSW/PVC and sTi–CSW/PVC composites.

Whisker Content (%)	0	5	10	20	30
CSW/PVC	70.3	70.4	73.0	74.3	74.4
sTi–CSW/PVC	70.3	73.8	74.4	73.8	72.9

**Table 3:** Degradation temperatures of PVC and its composites obtained from the TGA and DTG curves.

Sample	Temperature (°C)		
	T_onset_	T_rpd_	T_50_
Pristine PVC	276	285	314
CSW/PVC	277	286	325
sTi–CSW/PVC	277	285	325

The content of CSW in all composites is 5 wt. %.
